# Research Trends and Collaboration Patterns on Polymyxin Resistance: A Bibliometric Analysis (2010–2019)

**DOI:** 10.3389/fphar.2021.702937

**Published:** 2021-10-22

**Authors:** Alvaro Quincho-Lopez, Josmel Pacheco-Mendoza

**Affiliations:** ^1^ San Fernando Medical School, Universidad Nacional Mayor de San Marcos, Lima, Peru; ^2^ Bibliometrics Research Unit, Universidad San Ignacio de Loyola, Lima, Peru

**Keywords:** polymyxin resistance, colistin resistance, antimicrobial resistance, bibliometric, scopus

## Abstract

**Background:** Antimicrobial resistance is a serious public health problem that has become a global threat. Special attention should be given to polymyxins (polymyxin B and colistin) which, since their reintroduction into clinical practice, are considered “last resort” drugs. The objective of this study is to perform a bibliometric analysis of scientific research on polymyxin resistance.

**Methods:** Scopus was used to retrieve documents relevant to polymyxin resistance from 2010 to 2019. Data was exported to Microsoft Excel for table presentation. SciVal was used for volume and citation analysis as well as collaboration patterns. Also, we extracted data regarding the top documents, authors, countries, institutions, and the metrics of journals. VantagePoint and VOSviewer were used for geographical distribution of worldwide research and keyword co-occurrence analysis, respectively.

**Results:** A total of 1,409 documents were retrieved. The retrieved documents received 25.0 citations per document. Articles (73.88%) and letters (18.09%) were the most frequent types of documents. During 2010–2019, there was a significant growth in publications (*p*-value < 0.001). The received citations were 35,209 with a peak in 2016 (11,250 citations). China and the United States led the scientific production with 299 (21.2%) and 238 (16.9%) publications, respectively. Little or no contribution came from central Asia, Sub-Saharan Africa, and Latin America. Chinese institutions have caused the greatest impact, with *University of Zhejiang* (China) being the most prolific institution on the subject (88 documents). In terms of the most productive journals, *Antimicrobial Agents and Chemotherapy* ranked first with 196 (13.9%) documents. Most of the documents were published in quartile one journals and only had national collaboration (43.2%). Analysis of keyword co-occurrence revealed that research on polymyxin resistance during the last decade has focused on its relationship with public health, pharmacology, and genetics.

**Conclusion:** The number of documents on polymyxin resistance has increased significantly in the recent years, with a steep growth from 2016 onwards. China and the United States led the scientific production. Most of the documents were published in high-quality journals. Greater joint efforts and more contribution from central Asia, Sub-Saharan Africa, and Latin America are still needed to tackle this global problem.

## Introduction

Although efforts for the development of new antibiotics are multiple, their number is still insufficient, many of these are modifications of existing ones and only ensure temporary control (World Health Organization, 2017a). Meanwhile, antimicrobial resistance (AMR) continues to be one of the main problems in public health, not only because of the high cost at the social level but also because of the high economic cost, therefore urgent actions are necessary to face this global problem (Laxminarayan et al., 2013; Allcock et al., 2017; Luepke et al., 2017). The AMR, especially in gram-negative bacteria, has led to the rethinking of drugs that, due to their severe adverse effects, namely nephrotoxicity and neurotoxicity, were neglected more than 30 years ago (Falagas and Kasiakou, 2005). Such is the case of polymyxins, a group of polypeptide antibiotics composed of five different chemical structures (A-E), of which only polymyxin B and E have clinical relevance, the latter being better known as colistin (Azzopardi et al., 2013). Since their reintroduction into clinical practice, they are considered “last resort” drugs because they serve as a final alternative to the ineffectiveness of other drugs (Biswas et al., 2012).

In 2017, the World Health Organization (WHO) in the GLASS (Global Antimicrobial Resistance Surveillance System) report noted that, due to cases of carbapenem resistance, the use of colistin had increased significantly. Although cases of colistin resistance are rare in countries that have the possibility of monitoring it, an emerging resistance to this drug has been noted (World Health Organization, 2017b), especially in gram-negative pathogens (Srinivas and Rivard, 2017). Therefore the WHO developed a technical guide for the detection and reporting of colistin resistance (World Health Organization, 2018), in addition to molecular methods to support AMR surveillance (Rebelo et al., 2018; Borowiak et al., 2020). Since then, various efforts have been made by researchers to develop detection mechanisms for polymyxin resistance, mainly in Enterobacteriaceae (Nordmann et al., 2016b) and gram-negative bacteria (Nordmann et al., 2016a).

Bibliometrics is defined as the application of mathematical and statistical methods used to assess the quality and quantity of published scientific literature and to study research trends, citation analysis, authorship, impact of publications, journal analysis, as well as collaboration patterns in a certain field (Hood and Wilson, 2001; Van Raan, 2003). Previously, various drugs have been studied with bibliometric indicators (Sweileh et al., 2016b; Al-Jabi, 2017; Ramirez-Malule, 2018). AMR has also been studied in a general way (Sweileh and Moh’d Mansour, 2020) or individually, either focusing on which organ or system is affected, such as antimicrobial resistance to uropathogens (Sweileh et al., 2018), or according to the class of antibiotic, for example those resistant to carbapenem (Sweileh et al., 2016a), antiparasitics such as antimalarial drugs (Sweileh et al., 2017b), and antifungals such as triazoles (Sweileh W. M. et al., 2017). However, no bibliometric studies on polymyxin resistance have been performed, despite the interest that it could offer to researchers, clinicians, and pharmacists to know the evolution and the areas in which the research has focused in relation to the use of these drugs. Although colistin is the best-known example, it is preferred to study the class of antibiotics to which it belongs. Furthermore, there is evidence of cross-resistance between colistin and polymyxin B (Falagas and Kasiakou, 2005; Cai et al., 2015). Therefore, the objective of the current study is to assess the research trends and collaboration patterns of scientific publications on polymyxin resistance, and to identify the main contributors to the research evolution of this subject.

## Materials and Methods

### Database

Scopus (Elsevier BV Company, United States, available at: https://www.scopus.com/) was used to retrieve all relevant documents in the present study. Scopus is an international multidisciplinary database that has a greater number of journals in comparison to Pubmed or Web of Science, and is 100% inclusive of Medline (Falagas et al., 2008). In addition, Scival, the software used for calculating metrics for deeper analysis works hand in hand with Scopus since both were developed by Elsevier.

### Search Strategy

When performing the search query, different variants were extracted from the Medical Subject Headings (MeSH) from Pubmed and Emtree from Embase, and from a combination of both a complex search strategy was generated. A supplementary file shows this in more detail (see Supplementary Material 1).

The asterisk (*) was used as a truncator or wildcard to collect all the variants of the word that have a root in common. For example, when you enter resist*, the search engine will show results for both resist-ant and resist-ance. On the other hand, some documents, especially the oldest ones, reported their research with different versions of the same word, such as polymyxin, polymixin, etc. In order to retrieve the largest number of documents, vowels Y and I of “polymyxin” were replaced with the question mark (?), which allows replacing a single character, finally being written as pol?m?x?n. The W/2 was used to search for variants that have a maximum of two term or none among the searched words. For example, when searching for TITLE (colistin W/2 resist*), titles such as: colistin resistance, resistant to colistin, resistance to colistin, among others, will be retrieved.

Using the Scopus source feature, we limit the search to only “journals” as our source type. Erratum and articles in press were excluded. Results from 2020 to 2021 were also excluded to avoid delays related to indexation in Scopus, as at least 6 months is needed for Scopus metrics to be updated. Since our software (SciVal) can analyze the last decade, the study period was limited from 2010 to 2019. The validity of the search strategy was tested by manually reviewing retrieved documents and those false-positive results were excluded from the final search query.

### Data Analysis

The data was downloaded as a.csv file from Scopus and then was exported to Microsoft Excel 2016 for table and graphic presentation. The data retrieval time and analysis were on January 6, 2021. Information such as annual growth per year, language, subject area, and the type of publication were extracted directly from Scopus. For further analysis, the.csv file was exported to SciVal (Elsevier BV Company, United States, available at: https://www.scival.com/). The following bibliometric indicators were presented:

1) Number of documents and citations for the most productive countries, institutions, journals, and authors publishing scientific documents on polymyxin resistance.
2) Research collaboration for the top ten authors, countries, and institutions. Each publication is assigned to one of four mutually exclusive collaboration types, based on its affiliation information:
 • International collaboration: a study carried out by multiple authors/institutions from different countries.
• National collaboration: a study carried out by several authors/institutions from a single country.
• Institutional collaboration: a study carried out by several authors from the same institution in a single country.
• Single authorship: no collaboration research. A category that is added with the objective that the percentage of all categories add up to 100%. Data not shown in tables.

In addition, the research production of each country was adjusted according to the size of its population (https://www.cia.gov/library/publications/the-world-factbook/geos/ag.html). Spearman’s correlation coefficient was obtained through the STATA statistical package (version 15.0, StataCorp, College Station, TX, United States) to check the correlation between some variables (number of documents and number of citations, and the number of documents during the period of study) with a *p*-value ≤ 0.05 being statistically significant. Using SciVal, the number of documents per quartile was also counted according to Scimago Journal and Country Rank and CiteScore.

The concepts used for citation analysis and other metrics in the present study are defined as follows:

• Document count: it shows the number of publications an entity, country, journal, or author has indexed in Scopus.
• Citation count: it shows the total number of citations an entity, country, journal, or author has received since an item was published, up to the date of the last data cut.
• Citations per document: it indicates the average citation impact of each of an entity’s publications. It is calculated by dividing the citation count by the document count.
• CiteScore: calculates the average number of citations received in a calendar year by all items published in that journal in the preceding 3 years (Roldan-Valadez et al., 2019).
• Scimago Journal and Rank: It weights the value of a citation depending on the field, quality, and reputation of the journal that the citation comes from (Roldan-Valadez et al., 2019).

VOSviewer (version 1.6.10) was used to create a visual representation of the co-occurrence of the most relevant keywords (van Eck and Waltman, 2010). For these frequently encountered terms, a minimum of 200 occurrences was placed as a limit. A thesaurus was created to put singular and plural words together. Also, inter-country collaboration was presented using VOSviewer. VantagePoint software was used to graph the geographical distribution of worldwide publications on the topic. Before generating the graph, a process of disambiguation of the names of countries with accent was carried out. For example: México, Mexico; Perú, Peru; among others.

## Results

### Volume, Citation Analysis, Type of Research and Language

**FIGURE 1:**
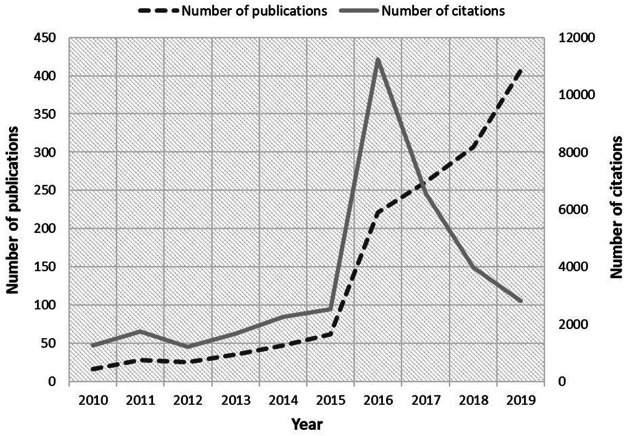
Growth of publications and citations for polymyxin resistance (2010–2019).

A total of 1,409 documents were retrieved. With 6,251 authors, there were 35,209 citations, with a peak recorded in 2016 (11,250 citations) and, in addition, an average of 25.0 citations/document were obtained. Figure 1 shows the number of documents and citations on polymyxin resistance. A significant increase in publications was observed during the period 2010–2019 (*r* = 0.9879, *p*-value < 0.0001), with a steep growth from 2016 onwards (>100 documents). Also, a high-level, positive, and statistically significant correlation was observed between the number of published documents and the number of citations (*r* = 0.8545, *p*-value = 0.0016). Annual distribution of documents per country and journal can be found in the Supplementary Material 2.

The 1,409 documents were published in 11 languages. English was the predominant language with 1,373 (97.4%) documents, followed very far by Chinese with 15 (1.1%) documents.

### Subject Areas of the Retrieved Documents

Most of the retrieved documents were published within the following subject areas (at least 100 publications): medicine (n = 1,124; 79.8%), pharmacology, toxicology, and pharmaceutics (n = 426; 30.2%), immunology and microbiology (n = 410; 29.1%), and biochemistry, genetics, and molecular biology (n = 189; 13.4%). It is important to note that a document can fit into one or more subject areas, therefore, the total percentages would exceed 100%.

### Collaboration Patterns of the Retrieved Documents

Most of the retrieved documents had only national collaboration (n = 606; 43.2%), followed by international collaboration (n = 483; 34.4%), and only institutional collaboration (n = 294; 21.0%). Nonetheless, in terms of impact, international collaboration (15,054 citations; 31.2 citations/document) exceeds both national (11,743; 19.4) and institutional collaboration (5,295; 18.0). The rest of the documents belong to the category “single authorship” or “no collaboration” (n = 26; 1.4%). Academic-corporate collaboration only represents 1.8% of total collaboration on polymyxin resistance research.

### Top Ten Cited Documents

The list of the 10 most cited publications on polymyxin resistance is presented in Table 1. Of these publications, two documents are reviews, one is a letter, and the rest are articles, within which is the most cited document on polymyxin resistance. This document was published in *The Lancet Infectious Diseases* and its number of citations quadruples those received by the second place.

**TABLE 1:** Top ten cited documents on polymyxin resistance (2010–2019).

Rank	Author	Title	Year	Source title	Citation	Type of document
1	Liu Y.-Y. et al.	Emergence of Plasmid-Mediated Colistin Resistance Mechanism MCR-1 in Animals and Human Beings in China: A Microbiological and Molecular Biological Study	2016	The Lancet Infectious Diseases	2,181	Article
2	Olaitan A.O. et al.	Mechanisms of Polymyxin Resistance: Acquired and Intrinsic Resistance in Bacteria	2014	Frontiers in Microbiology	546	Review
3	Cassini A. et al.	Attributable deaths and disability-adjusted life-years caused by infections with antibiotic-resistant bacteria in the EU and the European Economic Area in 2015: a population-level modelling analysis	2019	The Lancet Infectious Diseases	473	Article
4	Xavier B.B. et al.	Identification of a novel plasmid-mediated colistin resistance gene, mcr-2, in *Escherichia coli*, Belgium, june 2016	2016	Eurosurveillance	413	Article
5	Moffatt J.H. et al.	Colistin Resistance in *Acinetobacter baumannii* Is Mediated by Complete Loss of Lipopolysaccharide Production	2010	Antimicrobial Agents and Chemotherapy	411	Article
6	Cai Y. et al.	Colistin resistance of *Acinetobacter* baumannii: Clinical reports, mechanisms and antimicrobial strategies	2012	Journal of Antimicrobial Chemotherapy	328	Review
7	Tumbarello M. et al.	Infections caused by KPC-producing *Klebsiella pneumoniae*: Differences in therapy and mortality in a multicentre study	2015	Journal of Antimicrobial Chemotherapy	294	Article
8	Yin W. et al.	Novel plasmid-mediated colistin resistance gene mcr-3 in *Escherichia coli*	2017	mBio	282	Article
9	McGann P. et al.	*Escherichia coli* harboring mcr-1 and blaCTX-M on a novel IncF plasmid: First report of mcr-1 in the United States	2016	Antimicrobial Agents and Chemotherapy	246	Letter
10	Hasman H. et al.	Detection of mcr-1 encoding plasmid-mediated colistin-resistant *Escherichia coli* isolates from human bloodstream infection and imported chicken meat, denmark 2015	2015	Eurosurveillance	241	Article

### Top Ten Productive Countries

**FIGURE 2:**
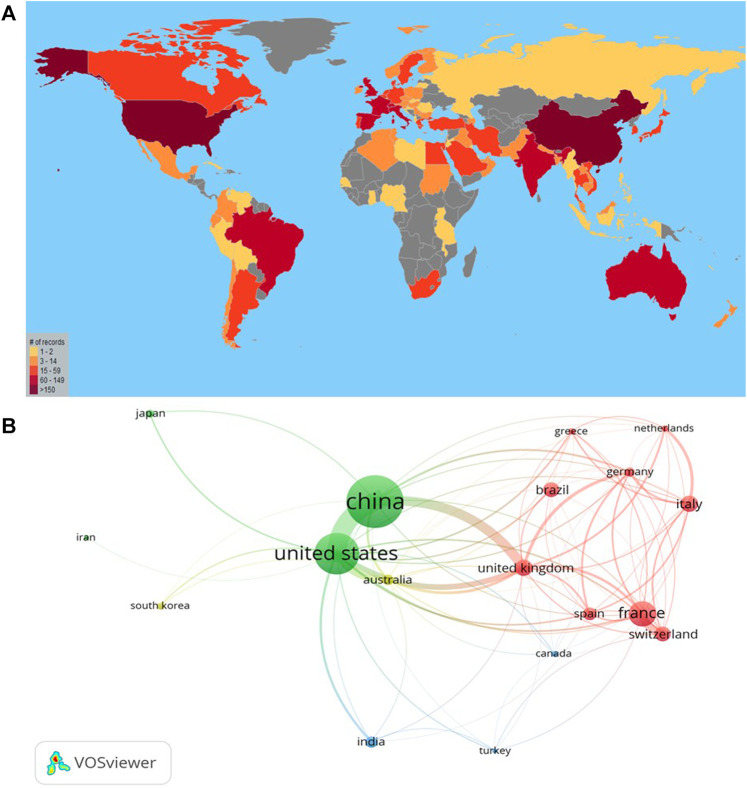
Spatial distribution of publications **(A)** Geographical distribution of worldwide publications on polymyxin resistance based on author’s affiliation in Scopus (2010–2019) **(B)** Network visualization of inter-country collaborations among countries with a minimum of 20 publications on polymyxin resistance research (2010–2019).

Among the 10 countries with the largest number of documents (Table 2), China is in first place with 299 documents and 8,405 citations, followed by the United States of America (United States) with 238 documents and 8,713 citations. It should be noted that the United Kingdom (United Kingdom) despite having fewer documents (n = 92) has the highest citation/document (n = 52.1). Figure 2A shows a geographical distribution of worldwide publications on polymyxin resistance using VantagePoint, in which darker colors indicate higher research activity while gray color indicates no contribution. Figure 2B shows inter-country collaborations among countries with a minimum of 20 publications on polymyxin resistance research using VOSviewer, in which the size of circles represents the number of publications of the country and the thickness of lines is in correlation with the size of collaboration between the countries.

**TABLE 2:** Top ten productive countries on polymyxin resistance (2010–2019).

Rank	Country	Documents N = 1,409 (%)	Documents/10 million inhabitants	Total citation	Citation/document	Collaboration (%)		
						International	National	Institutional
1	China	299 (21.2)	2.1	8,405	28.0	44.3	41.7	13.7
2	United States of America	238 (16.9)	7.2	8,713	36.6	66.0	19.7	13.9
3	France	146 (10.4)	21.8	4,511	30.9	63.7	31.5	4.8
4	Italy	94 (6.7)	15.2	3,057	32.5	37.2	42.6	20.2
5	United Kingdom	92 (6.5)	14.2	4,789	52.1	84.8	8.7	6.5
6	Switzerland	87 (6.2)	108.8	2,254	25.9	63.2	31.0	4.6
7	Brazil	85 (6.0)	4.0	1,065	12.5	16.5	54.1	23.5
8	Spain	74 (5.3)	14.8	1774	24.0	45.9	51.4	2.7
9	India	64 (4.5)	0.5	436	6.8	21.9	26.6	50.0
10	Australia	61 (4.3)	24.4	1704	27.9	77.0	8.2	13.1

We also calculated the field-weighted international and national collaboration (Supplementary Material 3) showing that most countries whose research is mainly based on international collaboration (>50%) obtain a greater global visibility.

### Top Ten Productive Authors

Table 3 shows the authors with the highest production on polymyxin resistance. *Nordmann P.* and *Poirel L.*, both with the same affiliation (*University of Fribourg*), dominate the list with the largest number of documents (58 and 56, respectively). Nonetheless, the most influential authors on polymyxin resistance were *Wang Y.* from China and *Doi Y.* from the United States with 3,471 and 3,312 citations, respectively.

**TABLE 3:** Top ten authors publishing on polymyxin resistance (2010–2019).

Rank	Author	Documents N = 1,409 (%)	Total citation	h-Index	Collaboration (%)			Country
					International	National	Institutional	
1	Nordmann, Patrice L.	58 (4.1)	1762	107	63.8	34.5	1.7	Switzerland
2	Poirel, Laurent	56 (4.0)	1816	101	62.5	35.7	1.8	Switzerland
3	Rolain, Jean Marc	45 (3.2)	1,557	59	53.3	44.4	2.2	France
4	Wang, Yang	41 (2.9)	3,471	38	58.5	34.1	7.3	China
5	Feng, Youjun	35 (2.5)	1,001	34	48.6	40.0	8.6	China
6	Li, Jian	32 (2.3)	1,111	58	75.0	9.4	15.6	China
7	Doi, Yohei	25 (1.8)	3,312	58	72.0	28.0	0	United States of America
8	Walsh, Timothy R.	22 (1.6)	2,934	65	95.5	4.5	0	United Kingdom
9	Sun, Jian	20 (1.4)	700	21	45.0	45.0	0	China
10	Ko, Kwan Soo	20 (1.4)	431	43	0	45.0	55.0	South Korea

### Top Ten Productive Institutions

The 10 institutions with the largest number of documents are shown in Table 4. The *Zhejiang University* (China), *University of Fribourg* (Switzerland), and *Center National de la Recherche Scientifique* (France) were the top three institutions with largest scientific production, respectively. However, only institutions from China (Zhejiang University, China Agricultural University and South China Agricultural University) have caused the greatest impact as they have the highest number of citations.

**TABLE 4:** Top ten productive institutions on polymyxin resistance (2010–2019).

Rank	Institution (country)	Documents N = 1,409 (%)	Total citation	Citation/document	Collaboration (%)		
					International	National	Institutional
1	Zhejiang University (China)	88 (6.2)	4,171	47.4	45.5	47.7	5.7
2	University of Fribourg (Switzerland)	59 (4.2)	1770	30.0	66.1	32.2	1.7
3	Centre National de la Recherche Scientifique (France)	53 (3.8)	1882	35.5	67.9	32.1	0
4	China Agricultural University (China)	47 (3.3)	3,506	74.6	59.6	34.0	6.4
5	Aix-Marseille Université (France)	46 (3.2)	1,532	33.3	54.3	43.5	2.2
6	Institut de Recherche pour le Développement (France)	44 (3.1)	1,505	34.2	54.5	45.5	0
7	Institut National de la Santé et de la Recherche Médicale (France)	42 (3.0)	1,596	38.0	57.1	42.9	0
8	South China Agricultural University (China)	41 (2.9)	3,350	81.7	43.9	39.0	17.1
9	Universidade de São Paulo (Brazil)	39 (2.8)	714	18.3	20.5	51.3	28.2
10	University of Lausanne (Switzerland)	38 (2.7)	749	19.7	73.7	26.3	0

### Top Ten Productive Journals

According to CiteScore Percentile, 582 (41.5%) documents on polymyxin resistance were published in the top 10% journals. The 10 journals with the highest number of publications on polymyxin resistance are shown in Table 5. The first three places were for *Antimicrobial Agents and Chemotherapy*, *Journal of Antimicrobial Chemotherapy,* and *International Journal of Antimicrobial Agents* with 196, 113 and 110 documents, respectively. However, only the first one maintains its place in terms of more citations (7,090 citations). Despite having few documents (n = 35), *The Lancet Infectious Diseases* obtained a significant number of citations and citation/document. The most common subject area of the top journals was Infectious diseases, followed by Microbiology and Pharmacology subject categories.

**TABLE 5:** Top ten journals publishing on polymyxin resistance (2010–2019).

Rank	Journal (country)	Documents N = 1,409 (%)	Total citation	Citations/document	Scimago journal and rank 2019	CiteScore 2019	Subject area category (quartile)^a^
1	Antimicrobial Agents and Chemotherapy (United States)	196 (13.9)	7,090	36.2	2.1	8.3	Infectious Diseases (Q1); Pharmacology (Q1); Pharmacology, Toxicology and Pharmaceutics (Q1)
2	Journal of Antimicrobial Chemotherapy (United Kingdom)	113 (8.0)	3,436	30.4	2.2	8.3	Infectious Diseases (Q1); Microbiology (Q1); Pharmacology (Q1); Pharmacology, Toxicology and Pharmaceutics (Q1)
3	International Journal of Antimicrobial Agents (Netherlands)	110 (7.8)	1985	18.0	1.5	6.7	Infectious Diseases (Q1); Medicine (Q1); Microbiology (Q1); Pharmacology (Q1)
4	Frontiers in Microbiology (Switzerland)	52 (3.7)	1,185	22.8	1.6	6.4	Microbiology (Q1); Immunology and Microbiology (Q1)
5	Journal of Global Antimicrobial Resistance (United Kingdom)	51 (3.6)	260	5.1	0.7	3.5	Microbiology (Q2); Immunology and Allergy (Q3); Immunology (Q3)
6	The Lancet Infectious Diseases (United Kingdom)	35 (2.5)	4,639	132.5	9.0	32.4	Infectious Diseases (Q1)
7	Microbial Drug Resistance (United States)	31 (2.2)	280	8.5	0.8	3.9	Immunology (Q3); Medicine (Q1); Microbiology (Q2); Pharmacology (Q2)
8	Diagnostic Microbiology and Infectious Disease (United States)	30 (2.1)	317	10.6	1.1	4.5	Infectious Diseases (Q2); Medicine (Q1); Microbiology (Q2)
9	Eurosurveillance (France)	30 (2.1)	2075	69.2	3.0	11.1	Epidemiology (Q1); Medicine (Q1); Public Health, Environmental and Occupational Health (Q1); Virology (Q1)
10	Infection and Drug Resistance (New Zealand)	28 (2.0)	194	6.9	0.9	2.4	Infectious Diseases (Q2); Pharmacology (Q2); Pharmacology, Toxicology and Pharmaceutics (Q1)

a Subject area according to Scimago Journal and Country Rank 2019.

Furthermore, according to CiteScore and Scimago Journal and Rank, Table 6 shows the number of documents sorted by the quartile of the journal in which they were published. The high concentration of these publications in quartile one journals suggests the high quality of the research.

**TABLE 6:** Number of documents classified by journal quartile according to CiteScore and Scimago Journal and Rank.

Classification system	CiteScore	Scimago journal and rank
Quartiles	N° Documents (%)	
Q1 (top 25%)	894 (63.4)	1,053 (74.7)
Q2 (26–50%)	260 (18.5)	181 (12.4)
Q3 (51–75%9	171 (12.1)	113 (8.0)
Q4 (76–100%)	77 (5.5)	51 (3.6)
No Q	7 (0.5)	11 (0.8)
Cumulative value	N° Documents (%)	
Q1 to Q2 (top 50%)	1,154 (81.9)	1,234 (87.6)
Q1 to Q3 (top 75%)	1,325 (94.0)	1,347 (95.6)

### Visualization of Research Themes

**FIGURE 3:**
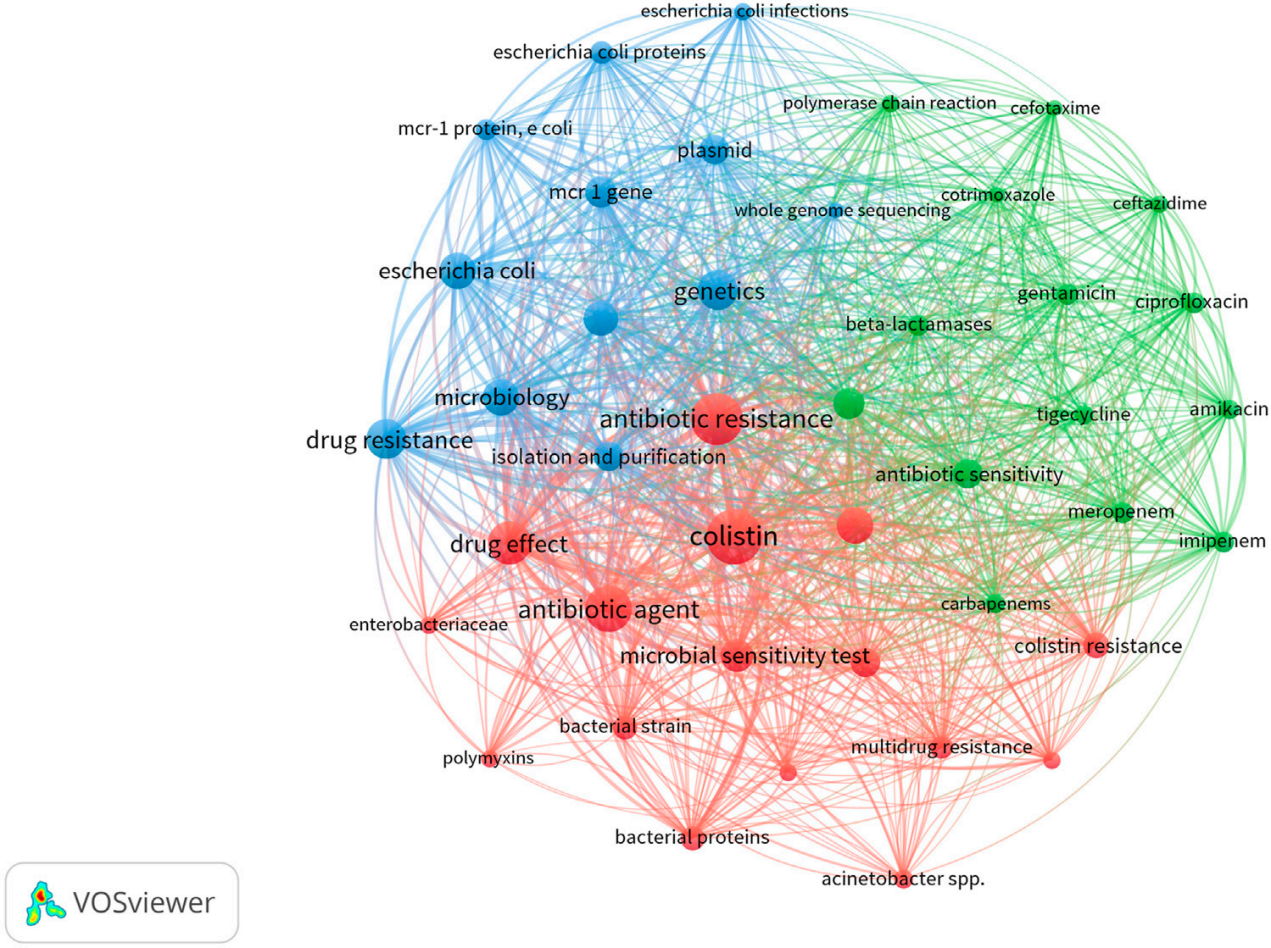
Research topics clustered by mapping of co-occurrences of terms for publications on polymyxin resistance (2010–2019). Of the 6,413 terms, 42 terms occurred at least 200 times. The size of the circles represents the occurrences of terms in title/key. The largest set of connected terms consists of 42 terms in three clusters. The three clusters may be interpreted as: “relationship of polymyxin resistance with public health (red cluster, 16 items)”, “other drugs ineffective against multidrug-resistant organisms including polymyxin-resistant pathogens (green cluster, 14 items)”, and “genetics of polymyxin resistance (blue cluster, 12 items)”.

Figure 3 shows visualization of terms on polymyxin resistance. Analysis of indexed keyword analysis also revealed that *Escherichia coli* (n = 674 occurrences), followed by *Klebsiella pneumoniae* (n = 499), and *Acinetobacter* spp. (n = 264) were the most frequently encountered pathogens.

### Trends for Clinical and Basic Research

**FIGURE 4:**
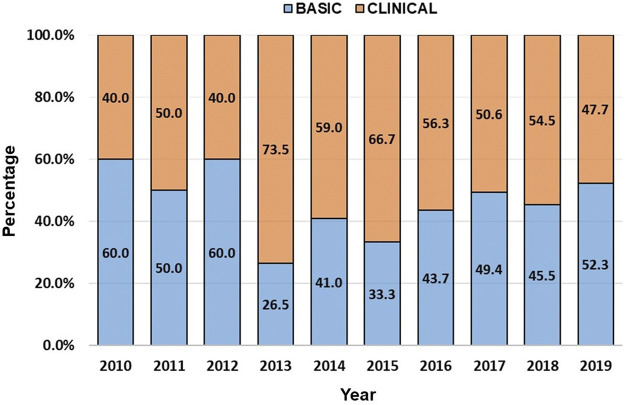
Basic and clinical research trends on polymyxin resistance (2010–2019).

For the manual classification of studies into basic and clinical research, secondary studies were excluded. Figure 4 shows the distribution of basic and clinical studies per year. Overall, while 47.2% (n = 599) of the studies were basic, the majority (52.8%; n = 669) consisted of clinical studies, although very few were randomized clinical trials. Similarly, clinical studies received more citations than basic studies (17,179 vs 13,815 citations).

## Discussion

In the present study, letters represent the second most frequent type of document after articles, unlike other bibliometric studies in Scopus on antimicrobial resistance among uropathogens and antimalarial drug resistance (Sweileh et al., 2017b, 2018), in which reviews obtain this position. However, only one letter is among the 10 most cited documents, with the rest being articles or reviews. An underlying explanation is that *The Lancet Infectious Diseases* and to a lesser extent other journals (*Antimicrobial Agents and Chemotherapy*, *International Journal of Antimicrobial Agents* and *Journal of Antimicrobial Chemotherapy*) accept research for publication under the category of research letter as a form of rapid communication, especially on relevant topics. An example of this is the most cited letter (246 citations) from McGann P. et al. (McGann et al., 2016) published in 2016 in *Antimicrobial Agents and Chemotherapy* and which deals with the first report of the mobilized colistin resistance (*mcr*-1) in the United States, found in an *E. coli* strain cultured from a patient with a urinary tract infection (UTI).

In 2016, the number citations increased dramatically (11,250 citations). This is probably due to the great impact that Liu Y-Y et al. (Liu et al., 2016) generated that year due to the discovery of plasmid-mediated resistance to colistin thanks to the *mcr*-1 gene identified in pigs in China, thus creating a paradigm shift in the resistance transfer mechanism, which was considered a rare chromosomal mutation until then. Interestingly, in the same year another gene called *mcr*-2 was isolated in *Escherichia coli* (*E.coli*) in Belgium (Xavier et al., 2016). In the following years other *mcr* genes were identified: in 2017 the *mcr*-3 gene in *E.coli* in China (Yin et al., 2017), *mcr*-4 in *E.coli* and *Salmonella enterica* serovar *Typhimurium* in three countries (Italy, Spain and Belgium) (Carattoli et al., 2017), *mcr*-5 in *E.coli* and *Salmonella Paratyphi* B in Germany (Borowiak et al., 2017), and *mcr*-6 in *Moraxella pluranimalium* in the United Kingdom (AbuOun et al., 2017); in 2018 the *mcr*-7.1 and *mcr*-8 gene, both in *Klebsiella pneumoniae* in China (Wang et al., 2018; Yang et al., 2018); in 2019 the *mcr*-9 gene in *Salmonella enterica* serovar *Typhimurium* in the United States (Carroll et al., 2019); and to date, the *mcr*-10 gene was isolated in *Enterobacter roggenkampii* in China (Wang et al., 2020). These have contributed to the continuous growth of the scientific literature relevant to polymyxin resistance, although they did not cause the same impact after 2016, as citations decreased (Figure 1). Given the wide availability of colistin compared to polymyxin B (Cai et al., 2015), it was expected that most of the literature would be on colistin resistance. This was partially confirmed by the 10 most cited publications (Table 3), as seven of them deal specifically with colistin resistance.

High-income countries were the largest contributors to the growth of the scientific literature. Unlike other bibliometric studies of drug resistance in which the United States ranked first in the amount of scientific production in resistance to other antimicrobials (Sweileh et al., 2016a; 2017b; Sweileh et al., 2017 W. M., 2018), the present study showed that China is the leading country on polymyxin resistance. Recently, a much more general bibliometric study focused on AMR in the environment showed that China is also leading scientific production (Sweileh and Moh’d Mansour, 2020). This may be partially explained by the fact that *mcr*-1 gene was first isolated in pigs in China (Liu et al., 2016). The increased antimicrobial consumption and the irrational use of antimicrobials in clinical settings and agriculture are the main reasons for AMR in China (Qu et al., 2019). Another key factor is that colistin is used for growth promotion in farm animals (Gharaibeh and Shatnawi, 2019). Indeed, colistin resistance among animals is a major concern in Asia and the Americas, ranging from 18 to 40%, and that China and India have the highest level of AMR in animals, and new hotspots of resistance are emerging in Brazil (Van Boeckel et al., 2019). In our study, Brazil and India ranked seventh and nineth, respectively. Also, a recent study demonstrated that China leads in terms of the numbers of worldwide reports made on *mcr* genes (4,917 strains identified in *mcr*-1 gene, and 274 strains identified in a variant *mcr* genes) (Elbediwi et al., 2019). The fact that more Chinese students and academics are collaborating with researchers from Western developed countries also contributes to China’s growth (Akanwa, 2015; Van Duin et al., 2018). As has been pointed out by other studies (Ramos-Rincón et al., 2019; González-Alcaide et al., 2020), China’s emergence in scientific production is remarkable and has advanced by leaps and bounds in recent years (Bornmann et al., 2015), including the area of pharmacology/pharmacy (Ding et al., 2013). Despite China’s positioning, the United States still maintains the highest number of citations (8,713 citations), a common finding in several similar studies (Sweileh et al., 2016a; 2016b; 2017b; 2017a). This is due to the international collaboration of the United States having a greater impact than that of China (field-weighted citation impact 1.47 vs 0.98) (see Supplementary Material 3), along with the consideration that citations usually have a delay of at least 2 years (Ding et al., 2013), and that China’s leading position recently emerged in 2017 (see Supplementary Material 2).

Given the large number of documents from China (n = 299) and the United States (n = 238), it is logical to expect them to obtain the highest number of citations (8,405 and 8,713, respectively). However, citation/document is a more reliable indicator, in which the United Kingdom ranks first with 51.2 citations per document. This may be due to their greater international collaboration (84.8%). When adjusted by population, some European countries, especially Switzerland, ranked higher. This along with their high number of citations may indicate that some small European countries publish high-quality documents. On the other hand, adjusting for population may not be the best approach for most densely-populated countries, so standardizing for the number of researchers in each country would be more realistic, although on issues like antimicrobial resistance where contributions may come from different areas, such information would not be easy to obtain (Zhao et al., 2015; Wang et al., 2017).

In the present study, the level of international collaboration (34.4%) was similar to population surveillance on *tuberculosis* (36.8%) or coronavirus (35.2%) (González-Alcaide et al., 2020) or high when compared to pneumonia (18.8%) (Ramos-Rincón et al., 2019), respiratory syncytial virus (13.3%) (Brüggmann et al., 2017) or Chagas cardiomyopathy (26%) (González-Alcaide et al., 2018). Although international collaboration is still not the most frequent type of collaboration, its importance is undeniable, making imperative the need for new strategies that facilitate its execution and implementation. As depicted in Figure 2, China and United States are the major contributors to this field. On the other hand, few or no contributions came from central Asia, Latin America, and Sub-Saharan Africa. The implementation of One Health strategies to combat Antibiotic Misuse in Low-and Middle-Income Countries (LMICs) includes, in addition to establishing adequate surveillance systems and strong laboratory capacity, also a multidisciplinary collaborative approach, in which the high-income countries support LMICs to overcome these cultural or socioeconomic barriers (Nadimpalli et al., 2018). Thus, international research collaboration is essential to improve the impact of a country’s scientific production (Wai-Chan, 2017; Aldieri et al., 2019). It is extremely important to establish collaboration networks between the Global North and South nations (Kim et al., 2017), since in this way the representativeness of the latter in global scientific production could be improved.

*Nordmann P.* and *Poirel L.*, who were the authors with the highest number of documents in polymyxin resistance, also have the highest number of documents in carbapenem resistance according to the bibliometric study carried out by Sweileh W. et al. (Sweileh et al., 2016a). This similarity may be explained by the fact that polymyxins are one of the few available options against carbapenem-resistant infections (Morrill et al., 2015), resulting in a unified investigation against multidrug resistant pathogens. Nonetheless, the most influential authors on polymyxin resistance were *Doi Y.* (United States) and *Wang Y* (China). The fact that both authors are co-authors of the most cited article would explain their high number of citations (see Table 3).

As for the 10 institutions with the highest scientific production, Chinese institutions were the most prolific in the topic. *Univeristy of Zhejiang* (China) was the institution whose publications caused the greatest impact (4,171 citations), with *Feng Y.* being the most productive author with this affiliation. It is also important to point out that, the *University o*f São P*aulo* (Brazil), while having a low international collaboration, ranked ninth among the most productive institutions (see Table 4). Its important national collaboration (51.3%) is noteworthy, being its greatest contribution on dissemination of the *mcr*-1 gene through samples collected in *E. coli* and other enterobacteria, evidencing an emerging resistance to colistin in the South American continent since 2012 (Fernandes et al., 2016).

*The Lancet Infectious Diseases* with only 35 documents achieved 132.5 citation/document. However, the most cited journal was *Antimicrobial Agents and Chemotherapy* (7,090 citations) and is the only one that has remained among the top five journals with the highest scientific production in other bibliometric studies, ranking first on carbapenem resistance (Sweileh et al., 2016a) and antifungal triazole resistance (Sweileh W. M. et al., 2017), second in antimalarial drug resistance (Sweileh et al., 2017b), and third in antimicrobial resistance among uropathogens (Sweileh et al., 2018).

When reading this article, it should be noted that it was not possible to discern between documents restricted to humans or animals. Nonetheless, the relationship is closer than it seems because colistin is used as a growth promoter in the veterinary field (Gharaibeh and Shatnawi, 2019). Even *mcr* genes were initially discovered in pigs in China (Liu et al., 2016). Furthermore, horizontal transmission of *mcr* genes occurs through multi-resistance plasmids from animals, and retail meat (Hasman et al., 2015; Gharaibeh and Shatnawi, 2019). However, this is consistent with the One Health approach, a concept that supports that health of humans, animals, and the environment are inextricably linked, and therefore should be recognized as one when combating AMR (Nadimpalli et al., 2018). In the present study, 20.3% of the documents deal with animals, 4.4% with food, and 2.7% with wastewater/sewage, thus recognizing once again the intrinsic relationship between the components of One Health.

## Limitations and Strengths

Finally, there are some limitations and strengths in our research. First, due to limitations of our software, only the last 10 years were analyzed. Thus, only the most recent publications (2010–2019) were included, which represent 90% of all documents available on the subject in Scopus. Second, like other bibliometric studies, some results may have been missing due to publication in non-indexed journals in Scopus. However, this is the first study on polymyxin resistance applying bibliometric indicators. Third, our study focused only on documents from journals and did not include grey literature that may have important information such as governmental or technical reports. We only analysed bibliometric data from the Scopus database, which may not reflect the complete set of research in the topic. Nevertheless, as the Scopus database only includes journals that meet high research standards, the dataset ensures that our results are based on documents that met those requirements and passed a strict peer-review process.

## Conclusion

The number of documents on polymyxin resistance in Scopus has increased in the last decade. Most of the documents were published by high-income countries, with China and the United States leading the scientific research activity on polymyxin resistance. This, together with the high-quality journals in which they were published, demonstrates the great importance of the subject and the rapid spread of AMR. The comparability of basic and clinical studies shows that much remains to be discovered on this subject. The discovery of the *mcr* gene caused great impact in the scientific community, attracting efforts from the fields of genetics, pharmacology, and public health. Greater joint efforts from researchers and clinicians in low- and middle-income countries with peers in high-income countries are needed to implement and carry out research on polymyxin resistance, establishing deep connections and increasing the levels of international collaboration.

## Data Availability

The original contributions presented in the study are included in the article/Supplementary Material, and further inquiries can be directed to the corresponding author.
