# Supplementary material for: Research Trends and Collaboration Patterns on Polymyxin Resistance: A Bibliometric Analysis (2010–2019)
Source: Front Pharmacol. 2021 Oct 22;12:702937. doi: 10.3389/fphar.2021.702937 (PMC8569321; doi:10.3389/fphar.2021.702937)
Supplement: Supplementary file 1 [file Table1.DOCX]

***Supplementary Material 1.***

**Search strategy on polymyxin resistance research (2010-2019)**

| **Steps** | **# Results** |
| --- | --- |
| **Combination of TITLE and KEY** | 2000 |
| ( ( ( TITLE ( colistin W/2 resist* ) ) OR ( TITLE ( colistimethate W/2 resist* ) ) OR ( TITLE ( colimycin W/2 resist* ) ) OR ( TITLE ( "pol?m?x?n E" W/2 resist* ) ) OR ( TITLE ( "pol?m?x?n B" W/2 resist* ) ) OR ( TITLE ( pol?m?x?n W/2 resist* ) ) OR ( TITLE ( "mcr-1" OR "mcr-2" OR "mcr-3" OR "mcr-3.1" OR "mcr-4" OR "mcr-4.3" OR "mcr-5" OR "mcr-6" OR "mcr-7" OR "mcr-7.1" OR "mcr-8" OR "mcr-8.1" OR "mcr-8.2" OR "mcr-9" OR "mcr-10" ) ) ) OR ( KEY ( "colistin resist*" OR "colistimethate resist*" OR "colimycin resist*" OR "polymyxin E resist*" OR "polymyxin B resist*" OR "polymyxin resist*" OR "polymixin resist*" ) ) ) OR ( TITLE-ABS-KEY ( "mobilized colistin resistance" OR "mobile colistin resistance" ) ) |  |
| **Excluding false positive results** | 1931 |
| AND NOT TITLE ( "colistin in multidrug" OR "colistin for multidrug" OR "colistin against carbapenem" OR "colistin for carbapenem" OR "colistin on carbapenem" OR "colistin susceptibility" OR "colistin against multidrug" OR "colistin on pandrug" OR "polymyxin B on antibiotic" OR "polymyxin B on carbapenem" OR "polymyxin-sensitive" OR "Polymyxin B against" OR "colistin on multidrug" OR "colistimethate for multidrug" OR "polymyxin B in multidrug" OR "colistin for drug-resistant" OR "colistin versus multidrug" OR "intrathecal colistin" OR arsenal OR "remaining choice" OR nanocrystalline ) |  |
| **Limiting search period** | 1784 |
| AND PUBYEAR > 2009 |  |
| **Limiting results to “journals”, and excluding “articles in press” and “errata”** | 1729 |
| AND ( LIMIT-TO ( SRCTYPE , "j" ) ) AND ( EXCLUDE ( PUBSTAGE , "aip" ) ) AND ( EXCLUDE ( DOCTYPE , "er" ) ) |  |
| **Excluding results from 2020 and 2021** | 1409 |
| AND ( EXCLUDE ( PUBYEAR , 2020 ) OR EXCLUDE ( PUBYEAR , 2021 ) ) |  |
