# Supplementary material for: Research Trends and Collaboration Patterns on Polymyxin Resistance: A Bibliometric Analysis (2010–2019)
Source: Front Pharmacol. 2021 Oct 22;12:702937. doi: 10.3389/fphar.2021.702937 (PMC8569321; doi:10.3389/fphar.2021.702937)
Supplement: Supplementary file 2 [file Table2.DOCX]

***Supplementary Material 2.***

**Annual contribution per country on polymyxin resistance research (2010-2019)**

| **Rank** | **Country** | **2010** | **2011** | **2012** | **2013** | **2014** | **2015** | **2016** | **2017** | **2018** | **2019** | **Total** |
| --- | --- | --- | --- | --- | --- | --- | --- | --- | --- | --- | --- | --- |
| 1 | China | 0 | 3 | 1 | 0 | 1 | 2 | 34 | 69 | 99 | 90 | 299 |
| 2 | United States | 6 | 5 | 3 | 9 | 6 | 13 | 37 | 54 | 46 | 59 | 238 |
| 3 | France | 0 | 3 | 3 | 4 | 7 | 12 | 28 | 20 | 33 | 36 | 146 |
| 4 | Italy | 0 | 3 | 1 | 6 | 9 | 10 | 11 | 13 | 24 | 17 | 94 |
| 5 | United Kingdom | 0 | 1 | 1 | 1 | 2 | 1 | 12 | 22 | 20 | 32 | 92 |
| 6 | Switzerland | 0 | 0 | 0 | 0 | 3 | 3 | 26 | 20 | 15 | 20 | 87 |
| 7 | Brazil | 0 | 0 | 1 | 2 | 2 | 2 | 15 | 18 | 18 | 27 | 85 |
| 8 | Spain | 0 | 3 | 2 | 4 | 7 | 7 | 8 | 12 | 14 | 17 | 74 |
| 9 | India | 0 | 1 | 0 | 2 | 2 | 2 | 11 | 8 | 12 | 26 | 64 |
| 10 | Australia | 1 | 2 | 3 | 1 | 3 | 3 | 7 | 12 | 16 | 13 | 61 |

**Annual contribution per journal on polymyxin resistance research (2010-2019)**

| **Rank** | **Journal** | **2010** | **2011** | **2012** | **2013** | **2014** | **2015** | **2016** | **2017** | **2018** | **2019** | **Total** |
| --- | --- | --- | --- | --- | --- | --- | --- | --- | --- | --- | --- | --- |
| 1 | Antimicrobial Agents and Chemotherapy | 4 | 7 | 7 | 10 | 12 | 11 | 45 | 44 | 28 | 28 | 196 |
| 2 | Journal of Antimicrobial Chemotherapy | 0 | 1 | 1 | 0 | 6 | 7 | 19 | 25 | 26 | 28 | 113 |
| 3 | International Journal of Antimicrobial Agents | 0 | 2 | 2 | 1 | 3 | 7 | 16 | 23 | 37 | 19 | 110 |
| 4 | Frontiers in Microbiology | 0 | 0 | 0 | 0 | 1 | 0 | 2 | 12 | 19 | 18 | 52 |
| 5 | Journal of Global Antimicrobial Resistance | 0 | 0 | 0 | 0 | 1 | 0 | 0 | 8 | 14 | 28 | 51 |
| 6 | The Lancet Infectious Diseases | 0 | 0 | 0 | 0 | 0 | 0 | 26 | 4 | 0 | 5 | 35 |
| 7 | Microbial Drug Resistance | 0 | 0 | 0 | 1 | 0 | 1 | 1 | 3 | 7 | 18 | 31 |
| 8 | Diagnostic Microbiology and Infectious Disease | 0 | 0 | 1 | 0 | 2 | 2 | 1 | 6 | 11 | 7 | 30 |
| 9 | Eurosurveillance | 0 | 0 | 1 | 1 | 1 | 1 | 11 | 9 | 4 | 2 | 30 |
| 10 | Infection and Drug Resistance | 0 | 0 | 0 | 0 | 0 | 0 | 0 | 3 | 8 | 17 | 28 |
