# Supplementary material for: Research Trends and Collaboration Patterns on Polymyxin Resistance: A Bibliometric Analysis (2010–2019)
Source: Front Pharmacol. 2021 Oct 22;12:702937. doi: 10.3389/fphar.2021.702937 (PMC8569321; doi:10.3389/fphar.2021.702937)
Supplement: Supplementary file 3 [file Table3.DOCX]

***Supplementary Material 3.***

**Field-weighted Citation Impact (FWCI) for international and national collaboration of the 20 most productive countries on polymyxin resistance research (2010-2019)**

| **Rank** | **Country** | **Number of documents** | **International collaboration (field-weighted)** | **National collaboration (field-weighted)** |
| --- | --- | --- | --- | --- |
| 1 | China | 299 | 0.98 | 0.94 |
| 2 | United States | 238 | 1.47 | 0.75 |
| 3 | France | 146 | 1.48 | 1.14 |
| 4 | Italy | 94 | 0.85 | 0.86 |
| 5 | United Kingdom | 92 | 1.89 | 0.72 |
| 6 | Switzerland | 87 | 1.51 | 1.12 |
| 7 | Brazil | 85 | 0.34 | 0.91 |
| 8 | Spain | 74 | 1.13 | 1.2 |
| 9 | India | 64 | 0.53 | 0.44 |
| 10 | Australia | 61 | 1.68 | 0.58 |
| 11 | Germany | 55 | 1.62 | 1.15 |
| 12 | Japan | 47 | 1.23 | 1.09 |
| 13 | South Korea | 42 | 0.36 | 0.68 |
| 14 | Greece | 37 | 1.06 | 0.76 |
| 15 | Netherlands | 36 | 1.32 | 1.29 |
| 16 | Canada | 34 | 1.07 | 0.67 |
| 17 | Iran | 30 | 0.35 | 1.01 |
| 18 | Turkey | 29 | 0.75 | 0.87 |
| 19 | Belgium | 27 | 2 | 0.93 |
| 20 | Sweden | 27 | 2.3 | 0 |

Interpretation:

Field-weighted Citation Impact (FWCI) is the ratio of the total citations received by the denominator’s output, and the total citations that would be expected based on the average of the subject field.

A Field-Weighted Citation Impact of:

- 1 means that the output performs just as expected for the global average.
- > 1 means that the output is more cited than expected according to the global average. For example, 1.48 means 48% more cited than expected.
- < 1 means that the output is cited less than expected according to the global average.
